# A Comparative Study on the Biosorption of Cd^2+^ onto *Paecilomyces lilacinus* XLA and *Mucoromycote* sp. XLC

**DOI:** 10.3390/ijms160715670

**Published:** 2015-07-10

**Authors:** Lu Xia, Xingjian Xu, Wei Zhu, Qiaoyun Huang, Wenli Chen

**Affiliations:** 1 State Key Laboratory of Agricultural Microbiology, Huazhong Agricultural University, Wuhan 430070, China; E-Mails: lovenicko0618@163.com (L.X.); xuxingjian@iga.ac.cn (X.X.); heiguangye@163.com (W.Z.); 2 Northeast Institute of Geography and Agroecology, Chinese Academy of Sciences, Changchun 130102, China; 3 Key Laboratory of Arable Land Conservation (Middle and Lower Reaches of Yangtze River), Ministry of Agriculture, College of Resources and Environment, Huazhong Agricultural University, Wuhan 430070, China

**Keywords:** filamentous fungi, Cd^2+^, biosorption, desorption, cadmium

## Abstract

The filamentous fungi XLA and XLC isolated from Cd-contaminated soil were identified morphologically and phylogenetically as *Paecilomyces lilacinus* and *Mucoromycote* sp., respectively. The minimum inhibitory concentrations (MICs) of Cd^2+^, Co^2+^, Cu^2+^, Zn^2+^, Cr^3+^ and Cr^6+^ in minimum mineral (MM) medium agar plates were 29,786, 2945, 9425, 5080, 1785 and 204 mg·L^−1^ for XLA and 11,240, 884, 9100, 2540, 3060 and 51 mg·L^−1^ for XLC, respectively. Favorable biosorption conditions for adsorption of Cd^2+^ by the tested fungi were investigated. Efficient performances of the biosorbents were described using Langmuir isotherm model, and the predicted maximum biosorption capacities for Cd^2+^ were 77.61 mg·g^−1^ of XLA and 79.67 mg·g^−1^of XLC. Experiments on desorption potential of biosorbents validated their efficacy at a large scale. Results showed that XLA obtained a desorption rate of 84.7% by 2% EDTA and XLC gained a desorption rate of 78.9% by 0.1 M HCl. Analysis by Fourier transform infrared spectroscopy (FTIR), scanning electron microscopy/energy dispersive X-ray spectroscopy (SEM/EDS) and X-ray photoelectron spectroscopy (XPS) suggested that groups of C–N, COO– for XLA and C–N, CH_2_ and phosphate for XLC were the dominant binding sites for Cd^2+^ biosorption. Our results indicated that the fungus XLA, rather than XLC, could potentially be used as an inexpensive, eco-friendly and effective bioremediation agent for the removal of Cd^2+^ from wastewater.

## 1. Introduction

Environmental pollution with toxic heavy metals is reaching critical degrees and becoming increasingly grievous with the development of industry and technology [1]. Heavy metals are non-biodegradable and tend to be accumulated in living organisms [2]. Cadmium, in the limelight among the toxic heavy metals due to its major impact on the environment, usually come from smelting, metal plating, batteries, mining, allay industries and sewage sludge [3]. The toxicity of cadmium includes a series of acute and chronic disorders, such as itai-itai disease, caused by the cadmium poisoning in Japan, testicular atrophy and hypertension [4,5]. Therefore, research efforts seeking an approach for the bioremediation of heavy metal contamination has become a great challenge [6,7].

Many conventional methods for dealing with heavy metals, such as chemical precipitation, evaporation and electrowinning, have been proved to be technologically inapplicable and costly from a comprehensive point of view [8,9]. What is worse, a large quantity of residual sludge from these processes might cause further pollution [10]. Recent research has focused on the biosorption of heavy metals by biological materials. This method is regarded as an economical, effective and eco-friendly technique for the remediation of heavy metal polluted environment [11,12].

Many microbial species, such as bacteria (*Bacillus substillis*), algae (*Sargassum* *natans*) and fungi (*Penicillium* *chrysogenum*), are known for their high metal biosorption capabilities [7,13]. The use of fungal biomass as a biosorbent has earned much attention because of its high percentage of cell wall material with excellent metal binding ability and its metal resistant properties [14]. Many filamentous fungi have proven potential for removing heavy metals, such as *Rhizopus*, *Penicillium*, *Aspergillus*, white rot fungi, *Mucor* and *Trichoderma* [15]. *Penicillium* *chrysogenum* was proved to possess the biosorption capability of 56.0 mg·g^−1^ for Cd^2+^ [16]. The maximum biosorption capabilities of *Lentinus* *edodes* for Hg^2+^, Cd^2+^ and Zn^2+^ are 336.3, 78.6 and 33.7 mg·g^−1^, respectively [17]. Moreover, filamentous fungi are ubiquitous in nature and easily available in substantial quantities, which could serve as an economic and constant supply of biosorbents [18]. Studies of the biosorption mechanism confirmed that the functional groups for the binding of metal ions are amid (–NH_2_), carboxylate (–COO), thiols (–SH), phosphate (PO_4_^3−^) and hydroxide (–OH) [7,19,20]. The adsorption stability depended mainly on the kind, amount, affinity and distribution of those groups [10]. Thus, biosorption of heavy metals by filamentous fungi can be suggested as a potential strategy for the remediation of Cd-contamination. However, the practical use of fungal biomass in field-scale remediation still needs further investigation. Thus, more fungal remediation agents need to be explored and biosorption mechanisms need to be complete.

The aim of the present investigation is to isolate cadmium-resistant filamentous fungi, measure their biosorption capabilities, and additionally analyze biosorption mechanism for their further practical applications.

## 2. Results and Discussions

### 2.1. Identification of Isolated Cd Resistant Fungi

Two morphologically different filamentous fungi were isolated by the selective medium under aerobic growing conditions from Cd-contaminated soil samples, which were named XLA and XLC, respectively. The morphological features of XLA and XLC on agar plates are shown in Figure S1. In brief, the colony of XLA was panniform, median rise and wine red, while colony of XLC was loose, villiform and white. The obtained sequences in the region between 18S and 28S rRNA genes of XLA (NCBI, GenBank accession no. HM004103) and XLC (NCBI, GenBank accession no. HM004102) were aligned with the sequences published in GenBank by BLAST. Sequence analysis of ITS1-ITS4 gene of the isolated fungi showed that strain XLA was similar to *Paecilomyces lilacinus* (NCBI, GenBank accession no. AM158216) with a similarity of 100% and strain XLC was similar to *Mucoromycotina* sp. FF67 (NCBI, GenBank accession no. FJ379796) with a similarity of 99%. According to morphological (Figure S1) and phylogenetic (Figure S2) analysis, fungal strains XLA and XLC were most closely related to *Paecilomyces lilacinus* and *Mucoromycote* sp., respectively.

### 2.2. Minimum Inhibitory Concentration (MIC) towards Heavy Metals

MICs of the tested heavy metals against XLA and XLC are listed in Table 1. These data indicated XLA and XLC possessing abilities to resist multi-metals, especially for Cd^2+^. In addition, the MIC data of Cr^6+^ suggested that XLA showed a remarkable difference under diverse cultivating conditions, because of various poisoning effects on the experimental growth stages. The variation of XLA and XLC in the heavy metal tolerance might be due to one or multiple kinds of resistant mechanism and tolerant strategies. Furthermore, differences in media composition along with the incubation time can affect the heavy metal complexation and bioavailability, thus leading to differences in metal tolerance [21]. In conclusion, the high resistant ability of XLA and XLC might indicate the potential ability to accumulate different metal ions and endure contaminated environment.

**Table 1:** Minimum inhibitory concentration of heavy metal against *P. lilacinus* XLA and *Mucoromycote* sp. XLC.

Strains	Cultivation Condition	Minimum Inhibitory Concentration (mg·L^−1^)					
		Cd^2+^	Co^2+^	Zn^2+^	Cu^2+^	Cr^3+^	Cr^6+^
XLA	Solid	29,786	2945	9425	5080	1785	204
	Liquid	34,844	2650	14,300	5080	3060	6375
XLC	Solid	11,240	884	9100	2540	3060	51
	Liquid	13,488	236	7800	8255	4335	51

### 2.3. Effect of pH on Biosorption

**Figure 1:**
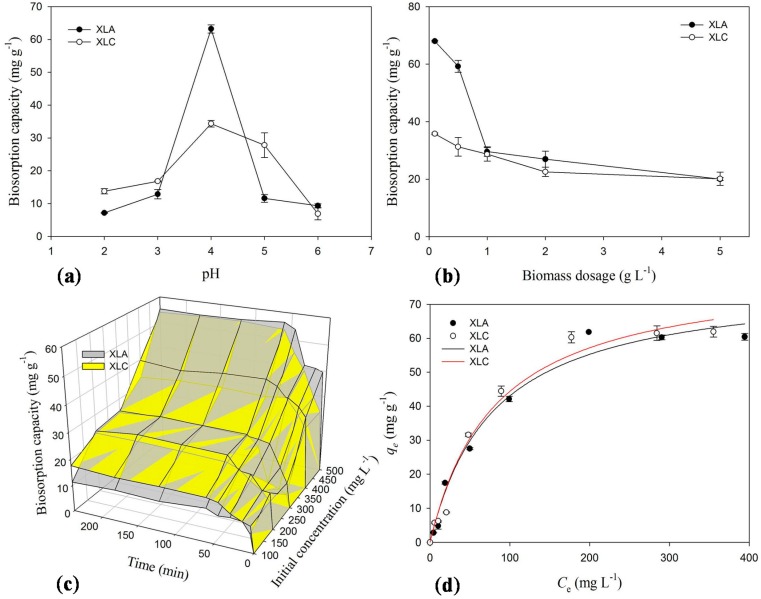
Effects of (**a**) pH; (**b**) biomass dosage; (**c**) contact time and metal concentration on biosorption capacity of *P. lilacinus* XLA and *Mucoromycote* sp. XLC for Cd^2+^; (**d**) Langmuir isotherm of *P. lilacinus* XLA and *Mucoromycote* sp. XLC for Cd^2+^ biosorption at optimum conditions. *q_e_* is the amount of metal ions adsorpted by unit biosorbents in mg·g^−1^, *C*_e_ is equilibrium concentration of metal solution in mg·L^−1^.The bars represent the standard deviation error of the mean (*n* = 3).

As depicted in Figure 1a, the removal of Cd^2+^ ions by biomass of both XLA and XLC were significantly influenced by solution pH. The adsorption capacity progressively increased up to pH 4, where the maximum biosorption ability was 63.22 mg·g^−1^ for XLA and 34.34 mg·g^−1^ for XLC, respectively. With the increasing of pH (pH > 4), the uptake of metal ions was seriously decreased to 9.33 mg·g^−1^ for XLA and 6.91 mg·g^−1^ for XLC. Therefore, all the further biosorption experiments for XLA and XLC were conducted at pH 4.

The competition of cadmium ions and protons in solution to the binding sites on the cell wall can explain the relationship between pH values and the uptake of metal ions [7]. At highly acidic pH, substantial amounts of H_3_O^+^ in the solution compete with metal ions for the binding sites on the cell surface, which leads to the lower uptake of metal ions. As the increase of pH, the negative charge density on the biosorbent surface increases on account of the deprotonation of active binding sites, and leads to the electrostatic attraction of some positively charged metals adsorbed on the biosorbents. On the other hand, the decrease of biosorption efficiency at higher pH value may attribute to the formation of aquo-complex of heavy metal ions surrounded by large number of hydroxyls which decrease the chance rate of metal ions combining with the functional binding sites and the dissolved metal ions concentration in the solution [22,23].

### 2.4. Effect of Biosorbents Dosage on Biosorption

The influences of biosorbent dosage on Cd^2+^ uptake for both XLA and XLC were investigated and the results are presented in Figure 1b. At the minimum dosage (0.1 g·L^−1^), the maximum adsorption ability was attained, which was 68 mg·g^−1^ for XLA and 35.8 mg·g^−1^ for XLC. With the increase of biomass dosage, the uptake of Cd^2+^ by XLA and XLC unanimously decreased to 20.0 and 20.16 mg·g^−1^, respectively. In conclusion, the optimum biomass dosage of XLA and XLC for further experiments was 0.1 g·L^−1^.

This phenomenon can be explained by “mass effect”, which means that aggregation of abundant biosorbents will reduce the effective surface area, which leads to the deficient use of functional sites on the cell surface for binding Cd^2+^ ions and the reduction of biosorption capacity. Similar trends have been reported for the biosorption of Cd^2+^ by *Penicillium chrysogenum* [7].

### 2.5. Effect of Initial Concentration and Contact Time on Biosorption

As shown in Figure 1c, after 4 h reaction, the adsorption capability increased from 11.29 to 55.31 mg·g^−1^ for XLA and from 17.62 to 54 mg·g^−1^ for XLC with the initial Cd^2+^ ions concentration ranging from 100 to 500 mg·L^−1^ after 4 h, respectively. On analysis, the optimum initial concentration of Cd^2+^ solution was 500 mg·L^−1^, because the maximum adsorption capacity of Cd^2+^ ions could be obtained. With the enhancement of the particle transfer forces by substantial ions in high concentration solution, the probability of collision between ions and cell surface and functional sites is greatly increased, which could lead to the improvement of metal uptake capability per gram biomass [7].

In addition, Figure 1c also indicated that the uptake of Cd^2+^ ions increased rapidly within 45 min for XLA and 60 min for XLC. The maximum adsorption for Cd^2+^ ions in solution occurred in 60 min for XLA and 120 min for XLC where the biosorption equilibrium was reached and the capacity was 56.34 and 54.66 mg·g^−1^, respectively. Thus, the optimum reaction condition of contact time for Cd^2+^ removal was at least 60 min for XLA and 120 min for XLC.

### 2.6. Biosorption Isotherms

The biosorption isotherm for Cd^2+^ by XLA and XLC biomass are shown in Figure 1d, and parameters are presented in Table S1. The high *r*^2^ values indicated that the Langmuir isotherm was well fitted to the experiment data for both XLA and XLC, which could be inferred that the key process is monolayer biosorption. The predicted maximum biosorption capacity was XLC (79.67 mg·g^−1^) > XLA (77.61 mg·g^−1^). The *K*_L_ value of 0.014 for XLA and 0.013 for XLC implied strong binding intensity in the biosorption process. Comprehensive inspection of the two constants *q*_max_ and *K*_L_ indicated that XLC was more efficient in adsorbing Cd^2+^ than XLA. The adsorption capacity of Cd^2+^ is much higher than the biosorbents used by other researchers, *i.e.*, 61.35 mg·g^−1^ for *Penicillium simplicissimum* [14], 18.55 mg·g^−1^ for *Botrytis cinerea* [24] and 27.79 mg·g^−1^ for *Phanerochaete chrysosporium* [25]. Therefore, we suggest that XLC biomass is a good alternative biomaterial for the heavy metal removal in environment, with a bit higher adsorption capacity than XLA biomass.

### 2.7. Desorption and Reuse

**Figure 2:**
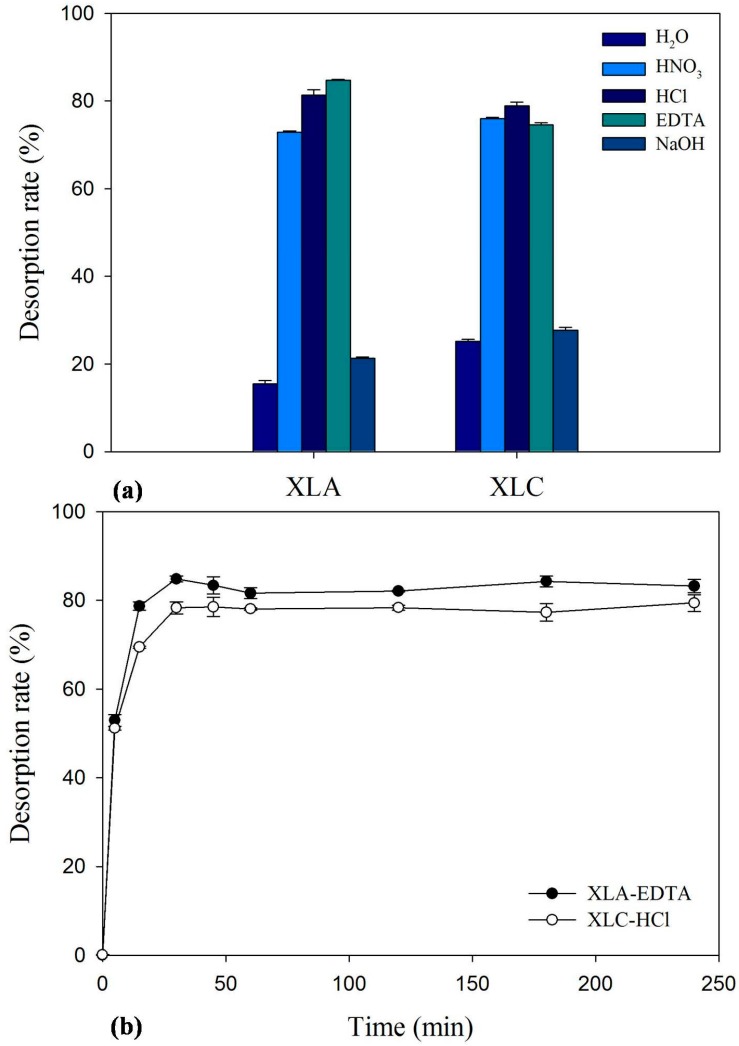
Desorption rate of different desorbing agents for (**a**) Cd-loaded *P. lilacinus* XLA/*Mucoromycote* sp. XLC; and (**b**) the time course of Cd^2+^ desorption from metal-loaded the tested biomass by 2% EDTA and 0.1 M NaOH, respectively. The bars represent the standard deviation error of the mean (*n* = 3).

To assess the potential of the isolated fungi for commercial application, the experiments were set up to select appropriate desorbents with high regeneration ability. The screening of effective desorbing agents was conducted and the results are presented in Figure 2a. Results obtained in this experiment revealed that Cd^2+^ is better desorbed with 2% EDTA for XLA and 0.1 M HCl for XLC, yielding a desorption rate of 84.7% and 78.9%, respectively. Figure 2b showed the results of desorption efficiency studies with optimum desorbing agent. The results indicated that desorption was a rapid process that the maximum desorption rate was obtained within 30 min for both XLA and XLC. With a high desorption rate and efficiency, it could be claimed that both XLA and XLC have a significant potential for biosorption and desorption of Cd^2+^. In addition, XLA is more likely to be a commercial and utilized potential biosorbents than XLC as its higher reusable efficiency by using low toxicity desorption agent.

### 2.8. Fourier Transform Infrared Spectroscopy (FTIR) Spectrum Analysis

**Figure 3:**
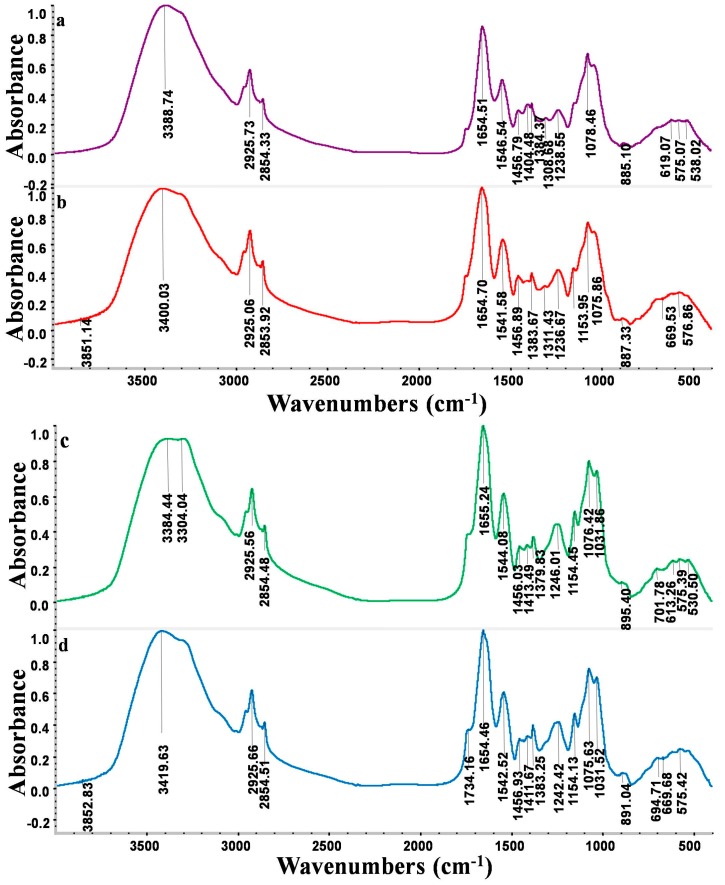
FTIR spectra of *P. lilacinus* XLA biomass (**a**) before and (**b**) after Cd^2+^ loaded; and FTIR spectra of *Mucoromycote* sp. XLC biomass (**c**) before and (**d**) after Cd^2+^ loaded.

The FTIR spectra of XLA and XLC before and after Cd^2+^ biosorption are presented in Figure 3. The main functional groups related to the strong peaks observed are identified by previous literature [7,26,27] and listed in Table 2.

**Table 2:** Main functional groups of Fourier transform infrared spectroscopy (FTIR) analysis.

Wavelength (cm^−1^)	Functional Group
3300–3400	–OH stretching vibration and –NH stretching of the protein
2925 and 2854	Asymmetric/symmetric stretching vibration of CH_2_
2364	Vibration of protonated amino (NH_2_^+^, NH^+^)
1739	Stretching vibration of C=O in carboxylic acids
1655	Stretching vibration of C=O and C–N (amide II) peptidic bond of protein
1545	Stretching vibration of C–N and deformation vibration of N–H (amide II) peptidic bond of protein
1404	Stretching vibration of carboxylate aminos (–COO) and deformation vibration of –OH
1380	Typical amide III band which is COO–aminos
1306	C–N stretching vibration of amino groups
1240	–SO_3_ groups
1078	C–N stretching vibration of amino groups
1152	Stretching vibration of C–O–C
884	Aromatic –CH stretching vibration
500–700 (fingerprint zone)	Phosphate or sulphate functional groups

Figure 3a,b showed that the broad stretching bands at 3389 and 1405 cm^−1^ strongly shifted to 3411 and 1419 cm^−1^ after Cd^2+^ loaded onto strain XLA, respectively. Another three peaks at 1654, 1546 and 1238 cm^−1^ were observed to become sharper after the loading of Cd^2+^. These obvious changes of adsorbance peaks were –OH, –NH, C=O, –NH deformation and –SO_3_ groups, which proved that acetamido groups, amide I, and sulfate could be active functional sites. The shoulder bands at 2925, 2854 and 1384 cm^−1^ became shaper. The bands at 1306 cm^−1^ disappeared and the peaks at 2364 cm^−1^ only appeared in the spectra of Cd^2+^-loaded biomass. These results indicated the presence of an interaction between Cd^2+^ and CH_2_, –NH_2_^+^, amino groups and amide III. The multiply adsorption peaks observed in fingerprint zone 500–700 cm^−1^ could be attribute to the interaction of between Cd^2+^ and phosphate and sulfate functional groups. Thus, studies of FTIR spectra suggested that –OH, COO–, NH_2_^+^ and C–N groups on the surface of XLA were significant functional sites for binding cadmium ions.

Figure 3c,d showed that adsorbance peaks strongly shifted from 3396 to 3419 cm^−1^, 1739 to 1745 cm^−1^, 1379 to 1383 cm^−1^ and 883 to 891 cm^−1^, which provided an evidence for the interaction of Cd^2+^ with –OH/–NH, –CH and amid III groups in biosorption of Cd^2+^ by strain XLC. On the other hand, the shoulder peaks at 2925, 2854, 1655 and 1245 cm^−1^ became sharper after Cd^2+^ loaded, which indicated the involvement of –CH_2_, amid I and –SO_3_ groups in the reaction with Cd^2+^. In addition, the disappearance of peaks at 709 and 529 cm^−1^ in fingerprint zone suggested the effectiveness of phosphate and sulfate groups in adsorption. Therefore, we concluded from the FTIR spectra that the key functional groups on the surface of XLC involved in the Cd^2+^ biosorption are –OH, CH_2_, –NH, C–N and phosphate groups.

### 2.9. Scanning Electron Microscopy/Energy Dispersive X-ray Spectroscopy (SEM/EDS) Analysis

**Figure 4:**
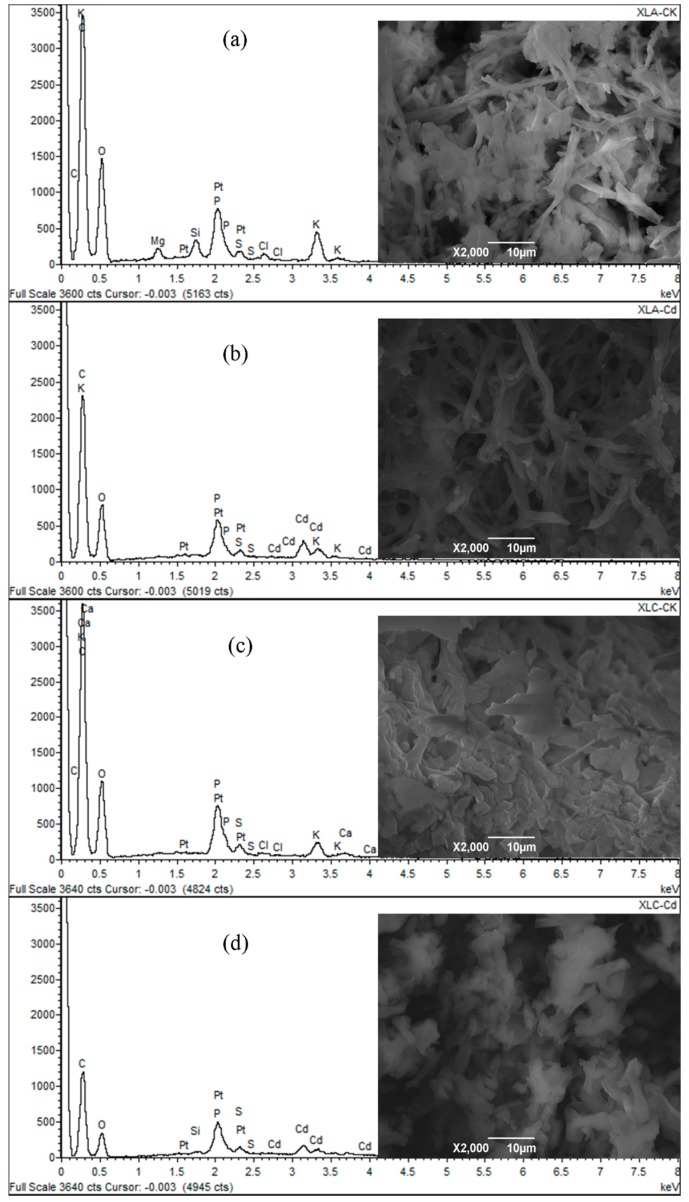
Typical scanning electron microscopy (SEM) micrograph and energy dispersive X-ray spectroscopy (EDS) spectrum of *P. lilacinus* XLA (**a**) before and (**b**) after Cd^2+^ loaded; and typical SEM micrograph and EDS spectrum of *Mucoromycote* sp. XLC (**c**) before and (**d**) after Cd^2+^ loaded.

The SEM micrographs and semi-quantitative analysis results of XLA and XLC biomass before and after biosorption are showed in Figure 4.

The micrographs in Figure 4b suggested the elongation and segregation of the mycelium and the appearance of some shinny small particles over the surface of Cd^2+^-loaded biomass of XLA, which were absent on the surface of the raw biomass (Figure 4a). The morphological alternation was explained by the EDS analysis, which revealed that the high intensity characteristic peak of Cd only exist after biosorption. Furthermore, the semi-quantitative analysis data listed in Table S2 proved that a large amount of Cd^2+^ was accumulated on cell surface after biosorption. In addition, the disappearance of Mg^2+^ peaks at 1.25 keV and the intensity decrease of K^+^ peaks at 0.3 keV and about 3.4 keV detected in EDS analysis, along with variation conservation of equivalent charges between Cd^2+^ and Ca^2+^/K^+^ surveyed in quantitative analysis completely indicated that Cd^2+^ is more competitive for the functional groups resulted in the ion-exchange process. The fact that the atomic percentage of P, S, and O increased proportionally with the addition of Cd ratio illustrated that the formation of precipitation in the form of sulfate and calcium phosphate on the cell surface, which was observed in SEM micrographs, was another probable biosorption mechanism. Therefore, we suggest ions-exchange mechanism and micro-precipitation mechanism play a crucial part in Cd^2+^ biosorption by XLA.

Similarly, according to Figure 4c,d, the coarseness and deformation of XLC were clearly observed with the loading of Cd^2+^. These morphological damages might be caused by Cd^2+^ adsorption. This speculation was also confirmed by EDS analysis which indicated the appearance of Cd^2+^ peaks at 2.7–3.8 keV evidently, only after Cd^2+^ biosorption. Moreover, the semi-quantitative analysis data presented in Table S3 also provided an evidence of the abundant accumulation of Cd^2+^ on the surface of XLC. The peaks of Ca^2+^ and K^+^ nearly disappeared and also could not be detected in quantitative analysis after biosorption, which proved the existence of ion-exchange. Generally, we concluded from the SEM/EDS analysis results that the biosorption of Cd^2+^ by XLC mainly depend on the ion-exchange mechanism.

**Figure 5:**
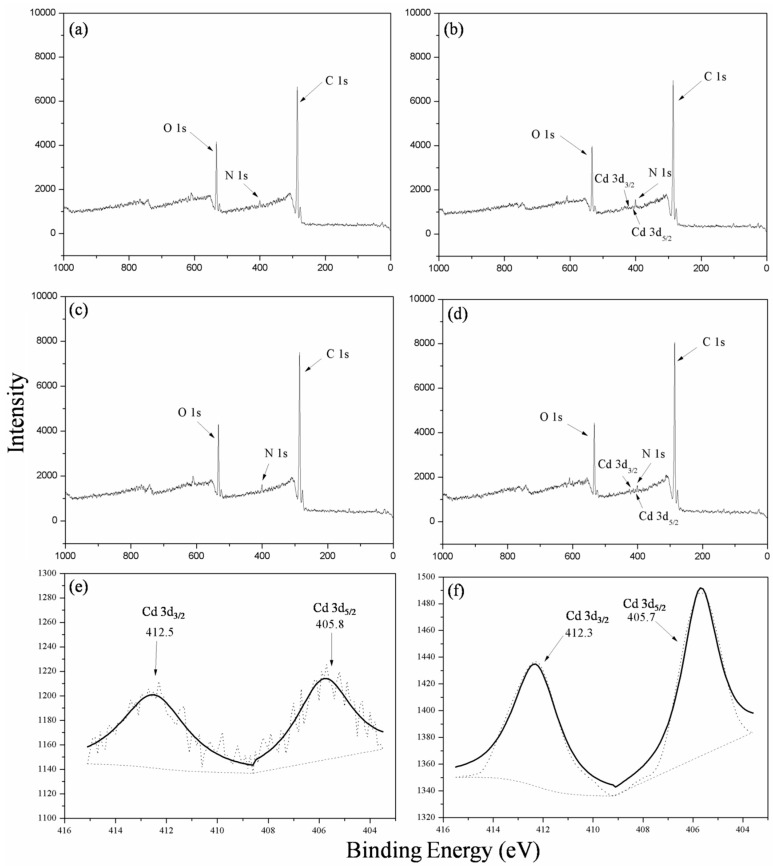
X-ray photoelectron spectroscopy (XPS) wide scanning spectra of *P. lilacinus* XLA (**a**) before and (**b**) after Cd^2+^ biosorption; XPS wide scanning spectra of *Mucoromycote* sp. XLC (**c**) before and (**d**) after Cd^2+^ biosorption; andthe deconvolution of Cd 3d spectra of (**e**) *P. lilacinus* XLA and (**f**) *Mucoromycote* sp. XLC after Cd^2+^ biosorption.

### 2.10. X-ray Photoelectron Spectroscopy (XPS) Analysis

The XPS scanning spectra of XLA and XLC biomass before and after Cd^2+^ biosorption are shown in Figure 5. The main elemental peaks detected were C 1s, N 1s and O 1s in all the XPS spectra. The results showed that the peaks of Cd did not exist in the XPS spectrum of the raw biomass, while after Cd adsorption, the peaks of Cd 3d_3/2_ and Cd 3d_5/2_ were present in the XPS wide scanning spectrum. The functional groups were characterized by the binding energy of C 1s and O 1s.

The changes of binding energy of the coordination carbon atom (C 1s) and oxygen atom (O 1s) in biosorbents XLA before and after Cd^2+^ biosorption were presented in Figure 5a,b, and the area ratio results were summarized in Table 3. The C 1s deconvolution spectra comprised three peaks with corresponding binding energy of 284.6, 285.2 and 286.3 eV, which could be assigned to C–C/C–H species, C–N/C–OR species and C–O component, respectively [7,28]. The area distribution of C 1s indicated that C–C and C–H would be the main carbon forms in XLA, and the form of C–C/C–H and C–O occupied nearly the same percentage. According to the data, the C–N/C–OR carbon ration was found to decrease after Cd-biosorption, indicating that the functional group interacted with Cd^2+^ might be C–N/C–OR species. The O 1s deconvolution spectra contained three peaks with the binding energy of 531.8 eV, C=O in the form of carboxylate; 532.8 eV, alcohol, hydroxyl and ether; and 533.7 eV, ester [29]. The decrease intensity (data not shown) of the peak 533.7 eV further revealed that the easter-cadmium complex might be formed on the biomass surface. The Cd 3d spectra comprised two peaks with corresponding binding energy of Cd 3d_3/2_ and Cd 3d_5/2_ states appeared around 405.8 and 412.5 eV, respectively, which were identified via the deconvolution. The presence of Cd 3d peaks after biosorption illustrated that the Cd^2+^ was successfully adsorbed by XLA by binding with the surface functional groups such as C–N, C–OR and ester species.

**Table 3:** Results of the deconvolution of C 1s and O 1s spectra for *P. lilacinus* XLA and *Mucoromycote* sp. XLC before and after Cd^2+^ biosorption.

Strain	C 1s			O 1s		
	Peak Position (eV)	Peak Area Ratio (%)		Peak Position (eV)	Peak Area Ratio (%)	
		Biomass without Cd	Biomass with Cd		Biomass without Cd	Biomass with Cd
XLA	284.6	46.13	48.48	531.8	34.66	33.91
	285.2	27.92	18.92	532.8	57.65	50.87
	286.3	25.95	34.60	533.7	7.70	15.22
XLC	284.6	38.66	45.36	532.0	43.65	42.46
	285.3	39.71	30.43	533.0	48.86	45.77
	286.6	19.19	21.62	533.9	7.49	11.47
	288.1	2.44	2.59			

Similarly, Figure 5c,d exhibited avariety of binding energy of the coordination carbon atom and oxygen atom in XLC before and after Cd^2+^ biosorption, and their area ratios are listed in Table 3. The C 1s spectra included four peaks with the binding energy of 284.6 eV, C–H/C–C species; 285.3 eV, C–N/C–OR species; 296.6 eV, C–O component; and 288.1 eV, C=O species [7,28]. The area ratio changes suggest that C–N/C–OR might be responsible for the binding of cadmium ions due to its decreasing carbon percentages. In addition, the result of the O 1s deconvolution spectra of XLC (Table 3) was similar to the result of XLA. The Cd 3d spectra contained two peaks with corresponding binding energy of Cd 3d_3/2_ and Cd 3d_5/2_ states appeared around 405.7 and 412.3 eV that were identified via the deconvolution, respectively. Thus, C–N/C–OR species could be regarded as the functional groups for Cd^2+^ biosorption of XLC.

The XPS analysis combining with the results of FTIR and SEM/EDS revealed that the functional groups of C–N, COO– for XLA and C–N, CH_2_ and phosphate for XLC could be considered as the significant binding sites in Cd^2+^ biosorption. These results helped us to acknowledge the biosorption mechanism for both *P. lilacinus* XLA and *Mucoromycote* sp. XLC.

## 3. Materials and Methods

### 3.1. Isolation of Cadmium-Resistant Filamentous Fungi

Cd-contaminated soil samples (50 mg·kg^−1^ Cd^2+^) collected from laboratory-scale pot experiments [7] were used for isolation of Cd resistant fungi. In brief, 10g of soil sample was suspended with 90 mL sterilized deionized water with five 0.5 mm sterilized glass beads and shaken on a rotatory shaker under aerobic condition at 200 rpm and 28 °C for 30 min. Ten-fold serial dilution of supernatant suspension was conducted to obtain desired solution (10^−3^). Varied diluted suspension samples (100 μL) were spread on selective Martin medium plates (1% glucose, 0.5% peptone, 0.05% MgSO_4_·7H_2_O, agar 2%, *m*/*v*) in the presence of 1686, 2248, 2810, and 5620 mg·L^−1^ Cd^2+^ (equal to 15, 20, 25 and 50 mM Cd^2+^) as screening condition, respectively. Control treatment with spreading 100 μL sterilized deionized water was carried out to avoid contaminating microorganisms. The inoculated plates were incubated at 28 °C for 5 days or more. The fungi with highest cadmium resistance were selected for purification and subcultured on Potato Dextrose Agar (PDA) medium [7]. In brief, purification process of the isolated fungi was as follows: mycelia of the isolated fungi were streaked inoculation on PDA plates using inoculating loop on a clean bench. The plates were incubated at 28 °C for 5 days to obtain fresh mycelia. Previous steps were repeated at least five times to achieve stain purification. The primary identification of morphology was carried out on the basis of plate colonies.

### 3.2. Molecular Identification of Cadmium-Resistant Filamentous Fungi

After 3 days of incubation in the Martin medium at 180 rpm and 28 °C, the fresh mycelia of the isolated fungi were harvested and washed with sterilized distilled water. The genomic DNA of the fungus was extracted by the method described by Xu *et al.* [7]. Sequences between 18S and 28S rRNA gene were amplified using the primers ITS1 (5ʹ-TCCGTAGGTGAACCTGCGG-3ʹ) and ITS4 (5ʹ-TCCTCCGCTTATTGATATGC-3ʹ) [7]. The polymerase chain reaction (PCR) was performed with a MyCycler™ thermal cycler (Bio-Rad, Hercules, CA, USA). PCR reaction mixtures (50 μL) consisted of 5 μL 10× PCR buffer (containing Mg^2+^), 2 μL10 mM dNTPs, 2 μL each of 10 μM ITS1 and 10 μM ITS4 primers, 0.5 μL Taq DNA Polymerase (TianGen Biotech, Beijing, China) and 1 μL genomic DNA as template. The PCR reaction program carried out as follows: 95 °C for 2 min, followed by 35 cycles of 96 °C for 30 s, 54 °C for 1 min and 72 °C for 1 min, subsequently, a final extension at 72 °C for 10 min. After electrophoresis analysis in 2.0% (*w*/*v*) agarose gels, the PCR amplified fragment was purified with AxyPrep™ PCR Cleanup Kit as described by the manufacturer (AxyGen, Biosciences, Union City, CA, USA), and then cloned into pTA2 vector system (ToYoBo, Tokyo, Japan). The products of ligation were transformed into *E. coli* DH5α competent cells. Transformants were selected based on the blue/white colony selection on LB agar plates containing 100 μg·mL^−1^ of ampicillin (Amresco, Solon, OH, USA) and X-Gal/IPTG (Amresco). Positive clones with correct ITS1-ITS4 sequence inserts were subsequently sequenced. The obtained sequences were deposited in the NCBI database.

### 3.3. Determination of Resistance to Varied Heavy Metals

The minimum inhibitory concentration (MIC) was measured to evaluate the resistance of isolated fungus to toxic heavy metals. MIC could be generally defined as the lowest antimicrobial concentration that will inhibit the visible growth of a microorganism after incubation. Stock metal solutions of Cd^2+^ (562,000 mg·L^−1^), Cu^2+^ (317,500 mg·L^−1^), Co^2+^ (294,500 mg·L^−1^), Zn^2+^ (325,000 mg·L^−1^), Cr^3+^ (51,000 mg·L^−1^) and Cr^6+^ (51,000 mg·L^−1^) were added separately to minimum mineral medium (MM: 5 g (NH_4_)_2_SO_4_, 0.6 g MgSO_4_, 0.6 g CaCl_2_, 0.005 g FeSO_4_·7H_2_O, 0.002 g CoCl_2_ and 20 g glucose per liter medium with a low phosphate content 1.5 g KH_2_PO_4_, pH 5.5) to obtain the desired metal concentration, respectively. Firstly, 8 heavy metal levels (the metal concentrations (mg·L^−1^) were equal to 1, 10, 50, 100, 150, 200, 300 and 400 mM for each metal species) were tested to inhibit fungal growth. According to the observation of fungal growth, appropriate inhibitory metal levels were speculated and the final metal concentrations were adjusted with increase or decrease at an interval of 1 or 10 mg·L^−1^ in the predicted range. Finally, the MICs of heavy metals against the tested fungi were obtained. The experimental procedure in details was as follows: Under liquid culture condition, the MIC values were determined by the observation of colonial growth in the MM medium containing heavy metals. After germination rate was up to 80%, spores were collected by centrifugation at 5000 rpm for 10 min, washed with sterile distilled water and resuspended to a density of 10^6^ spores·mL^−1^. Thereafter, 50 μL of spore suspension was added into 20 mL flask containing 5 mL of MM medium with desired heavy metal concentration. After 7 days of inoculation at 180 rpm and 28 °C, the fungal biomass was measured. On agar plates of MM medium, MIC was calculated by the changes of colony diameters. Each plate was trisected, and the mycelium pellet of the same diameter obtained from MM liquid cultures was spot planted in triple on plates and then inoculated at 28 °C for 7 days. The survey of colony diameters each day was important index for MIC. Analysis of the diameter changes could provide information about the critical concentration to inhibit the growth of mycelium pellet, which can be regarded as MIC values.

### 3.4. Batch Biosorption Studies

Spores of the isolated fungi were grown in the MM medium at pH 5.5. After appropriate incubation of XLA and XLC at 180 rpm and 28 °C for 7 days, the culture was harvested by filtration using Whatman No. 11 filter paper, rinsed abundantly with distilled water and finally with distilled deionized water in order to remove adhering debris and culture medium. The samples were freeze-dried overnight, scattered softly, and homogenized for further biosorption experiments.

The standard stock heavy metal solutions (analytical grade) containing 500 mg·L^−1^ Cd were prepared by dissolving 1.372 g Cd(NO_3_)_2_·4H_2_O with 1000 mL distilled deionized water. Working solutions were prepared by diluting the stock solution with distilled deionized water. All batch biosorption experiments were carried out in 50 mL Erlenmeyer flasks with 20 mL Cd^2+^ solution on an orbital shaker at 150 rpm and 28 °C. Control treatment without introduction of fungal biomass was set up. The metal concentration was detected by using Atomic Absorption Spectrophotometer (AAS, F-240, VARIAN, Palo Alto, CA, USA). All the experiments were conducted in triplicate.

The influence of pH on biosorption capacity was investigated by equilibrating the biosorption mixture containing both the biosorbents and heavy metal solutions (500 mg·L^−1^) in the pH range 2.0−6.0 for Cd^2+^. The initial pH for the Cd^2+^ solution was adjusted to desirable pH with 1 M NaOH and 1 M HCl. The biosorption mixture containing 0.1 g·L^−1^ freeze dried biosorbents was shaken at 150 rpm and 28 °C for contact periods up to 240 min, which was enough for biosorption equilibrium. Similarly, the effects of biosorbent dosage (0.1, 0.5, 1.0, 2.0, and 5.0 g·L^−1^) on biosorption were investigated. In addition, a set of experiment on initial concentration of Cd^2+^ ranging from 100 to 500 mg·L^−1^ was conducted. The samples were withdrawn at the time intervals of 0, 5, 15, 30, 45, 60, 120, 180 and 240 min and filtered through 0.25 μm filters. The residual Cd^2+^ ion concentration of filtrated samples was measured by AAS. The Cd^2+^ biosorption capacity of the tested fungi was calculated according to the follow equation:

*q_e_* = *v*·(*C*_0_ − *C*_e_)/*m* (1)

where *q_e_* (metal uptake) is the amount of metal ions adsorbed by unit biosorbents in mg·g^−1^, *v* is the volume of metal solution in the reaction system in mL, and *C*_0_ and *C*_e_ are the initial and equilibrium concentration of metal ions in the solution in mg·L^−1^, respectively. *m* is the dosage of added biosorbents in g.

### 3.5. Biosorption Isotherm

The biosorption isotherm carried out at the optimum experimental condition is the initial experimental step to determine the feasibility of treatment and whether the further research work should be continued. The equilibrium studies are conducted by changing the initial concentrations in the range of 0–400 mg·L^−1^ at respective optimum pH for 4 h, and the data were analyzed and described by Langmuir isotherm:

(2) $$q_{\text{e}}=\;\frac{q_{\text{max}}K_{\text{eq}}C_{\text{e}}}{1+K_{\text{eq}}C_{\text{e}}}$$

where *q*_e_ is the amount of metal ions adsorpted by unit biosorbents in mg·g^−1^, *C*_e_ is equilibrium concentration of metal solution in mg·L^−1^, *q*_max_ is the academic maximum amount of metal ions adsorbed per gram biosorbents in mg·g^−1^ and *K*_eq_ is Langmuir equilibrium constant related to the biosorption energy in L·mg^−1^.

### 3.6. Desorption Studies

The adsorbed Cd^2+^ (500 mg·L^−1^) by the biosorbents for equilibrium biosorption was eluted by several desorption agents such as distilled H_2_O, 0.1 M HNO_3_, 0.1 M HCl, 2% EDTA, 0.1 M NaOH. The flasks were shaken at 150 rpm and 28 °C for 240 min, thereafter the biosorbents were isolated by centrifugation and the eluted metal ions in the supernatant were measured by AAS. After verifying the favorable desorbing agents, the desorption efficiency of these agents for metal-loaded biomass was studied by measuring metal concentration of eluted solutions at desired time intervals (0, 5, 15, 30, 45, 60, 120, 180 and 240 min). Each experiment was set up in triplicate.

### 3.7. FTIR Studies

Fourier Transform Infrared Spectroscopy (NICOLET 5700, Thermo Nicolet, Waltham, MA, USA) study was conducted to investigate the functional groups of the biosorbents before and after Cd^2+^ biosorption. Samples were freeze-dried overnight after biosorption of Cd at 150 rpm and 28 °C for 4 h. The metal loaded and the raw samples were mixed with KBr in the proportion of 1:100 and compress to slice with high pressure, respectively. Infrared spectral were recorded in the region of 400–4000 cm^−1^ and the background information from the scan of only KBr was automatically removed from the sample data.

### 3.8. SEM/EDS Studies

Scanning electron microscopy and energy dispersive spectroscopy (SEM/EDS) analysis was employed to evaluate the effect of metal ions on the morphological property of fungal cell surface and further determine the type of metal ions presented on the fungal cell wall. The metal-loaded and the raw samples were coated with a layer of platinum and examined by SEM (JSM-6390LV, JEOL, Tokyo, Japan)/EDS (OXFORD INCA x-sight, Abingdon, Oxfordshire, UK).

### 3.9. X-ray Photoelectron Spectroscopy (XPS) Studies

The X-ray photoelectron spectroscopy (XPS, KSAM800, Kratos Ltd., Manchester, UK) analysis before and after metal biosorption was carried out to investigate the elemental composition of biosorbents. Monochromatic Mg Kα radiation (1253.6 eV) operated at 12.5 Kv and 16 mA was used as an energy source to record the XPS spectra. The system was operated at a base pressure of 2 × 10^−8^ mbar. The calibration of the binding energy of the spectra was performed with the C 1s peak of the aliphatic carbons, which is at 284.6 eV. The XPS spectra were decomposed into subcomponents and analyzed by XPSpeak 4.1 software package.

### 3.10. Statistical Analysis

Each experiment was repeated in triplicate. All data analyses were performed using an SPSS 13.0. The data were evaluated by ANOVA compared using Tukey’s test with significance set at *p* < 0.05. All plots were made using SigmaPlot (version 10.0, Systat Software, Inc., San Jose, CA, USA).

## 4. Conclusions

*P. lilacinus* XLA and *Mucoromycote* sp. XLC were isolated from Cd-polluted soil. The MIC results showed their high tolerance to various heavy metals. A series of biosorption and desorption experiments demonstrated their high capability to remove Cd^2+^ and potential to be used as biosorbents for extensive utilization. Through comprehensive comparisons, we suggested that XLA seemed more suitable to be used as a biosorbent, due to its high level of resistance, short equilibrium time, and high recovery rate during desorption, despite slightly lower biosorption capacity compared to XLC. The biosorption mechanism was illustrated by FTIR, SEM/EDS and XPS analysis, which showed groups of C–N and COO– for XLA and C–N, CH_2_ and phosphate for XLC are closely related with the binding of cadmium ions.

## Supplementary Materials

Supplementary materials can be found at http://www.mdpi.com/1422-0067/16/07/15670/s1.
