# Supplementary material for: A Comparative Study on the Biosorption of Cd2+ onto Paecilomyces lilacinus XLA and Mucoromycote sp. XLC
Source: Int J Mol Sci. 2015 Jul 10;16(7):15670–87. doi: 10.3390/ijms160715670 (PMC4519919; doi:10.3390/ijms160715670)
Supplement: Supplementary file 1 [file ijms-16-15670-s001.pdf]

Supplementary Information

Table S1. Isotherm model constants and regression coefficients for biosorption of Cd^{2+} by *Paecilomyces lilacinus* XLA and *Mucoromycote* sp. XLC from aqueous solution.

Strain	Experiment	Langmuir Parameters		
	q [a]	q_{max} [b]	K_L	r^2
XLA	61.89 ± 0.98	77.61 ± 0.58	0.014	0.981
XLC	61.94 ± 1.57	79.67 ± 1.14	0.013	0.979

[a,b] data was presented as mean value ± standard deviation (SD) (n = 3).

Table S2. Quantitative results of EDS analysis of XLA before and after Cd^{2+} loading.

Biomass Type	Elements	wt. (%)	Atomic (%)
XLA-CK	O	71.12 ± 0.70	86.36
	Mg	2.57 ± 0.19	2.05
	Si	2.90 ± 0.15	2.01
	P	6.99 ± 0.25	4.38
	S	1.53 ± 0.15	0.92
	Cl	1.09 ± 0.13	0.60
	K	5.79 ± 0.18	2.88
	Pt	8.01 ± 0.69	0.80
	Totals	100.00	100.00
XLA-Cd	O	70.67 ± 1.00	90.51
	P	7.55 ± 0.33	5.00
	S	1.65 ± 0.20	1.06
	K	0.88 ± 0.20	0.46
	Cd	12.41 ± 0.56	2.26
	Pt	6.84 ± 0.93	0.72
	Totals	100.00	100.00

Table S3. Quantitative results of EDS analysis of XLC before and after Cd^{2+} loading.

Biomass Type	Element	wt. (%)	Atomic (%)
XLC-CK	O	73.88 ± 0.87	89.38
	P	8.54 ± 0.32	5.33
	S	2.47 ± 0.21	1.49
	Cl	0.74 ± 0.16	0.41
	K	4.07 ± 0.20	2.01
	Ca	0.93 ± 0.15	0.45
	Pt	9.37 ± 0.86	0.93
	Totals	100.00	100.00
XLC-Cd	O	59.33 ± 1.46	85.56
	Si	0.87 ± 0.24	0.71
	P	9.91 ± 0.52	7.38
	S	2.89 ± 0.36	2.08
	Cd	12.39 ± 0.84	2.54
	Pt	14.62 ± 1.42	1.73
	Totals	100.00	100.00

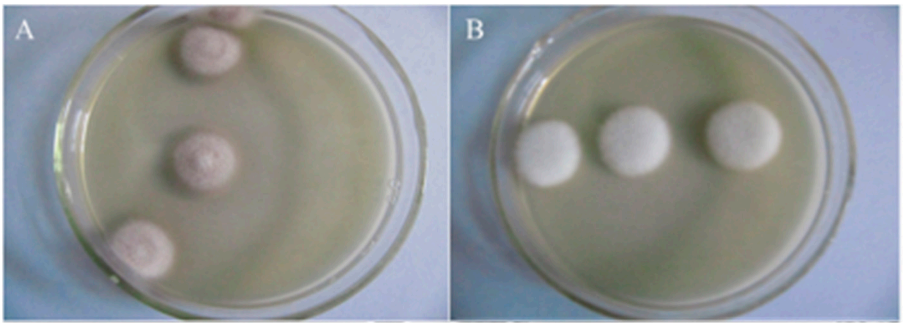

Figure S1. Colonial features of filamentous fungi (**A**) XLA and (**B**) XLC in potato dextrose agar (PDA) medium.

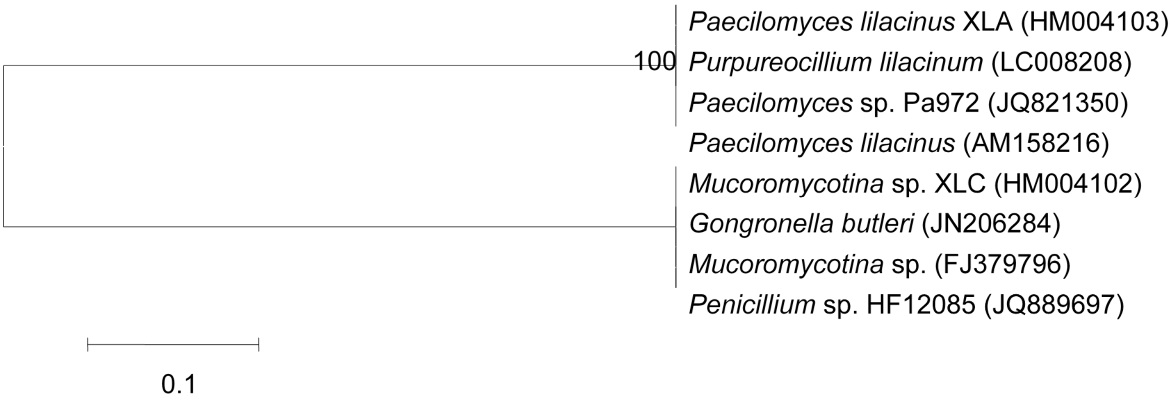

Figure S2. Neighbor-joining phylogenetic tree based on a comparison of the ITS1-ITS4 gene sequences of strain XLA, XLC and their closest relative strains. Bootstrap values equal to or greater than 50% are shown, and the scale bar represents the number of substitutions per site. The accession number is shown directly next to the genera name.
